# Analysis of the Mediating Role of Cytokines in the Relationship Between Nonalcoholic Fatty Liver Disease and Cognitive Impairment After Ischemic Stroke

**DOI:** 10.1002/brb3.71725

**Published:** 2026-09-28

**Authors:** Xin Du, Jingwei Li

**Affiliations:** ^1^ Department of Neurology Suzhou Hospital, Affiliated Hospital of Medical School, Nanjing University Suzhou China; ^2^ Department of Neurology Affiliated Drum Tower Hospital of Nanjing University Medical School Nanjing China; ^3^ Suzhou Research Centre of Medical School Nanjing University Suzhou China; ^4^ Department of Neurology, Medical School and the State Key Laboratory of Pharmaceutical Biotechnology, Drum Tower Hospital Nanjing University Nanjing China; ^5^ Institute of Brain Sciences Nanjing University Nanjing China; ^6^ Jiangsu Key Laboratory for Molecular Medicine Medical School of Nanjing University Nanjing China; ^7^ Jiangsu Province Stroke Center for Diagnosis and Therapy Nanjing China; ^8^ Nanjing Neurology Clinic Medical Center Nanjing China

**Keywords:** cytokines, nonalcoholic fatty liver disease, poststroke cognitive impairment

## Abstract

**Objective:**

To explore whether nonalcoholic fatty liver disease (NAFLD) is associated with poststroke cognitive impairment (PSCI) and to investigate the mediating role of cytokine levels.

**Research methods:**

This study included 247 patients with ischemic stroke. The subjects were divided into no‐to‐mild and moderate‐to‐severe groups according to the severity of NAFLD. The levels of peripheral blood cytokines and the scores of the Montreal cognitive assessment scale (MoCA) were measured for all subjects. The scale scores and cytokine levels were compared between the two groups, and the correlations among NAFLD, cytokines, and cognitive function were analyzed.

**Results:**

Compared with those in the no‐to‐mild group, the levels of IL‐1β, IL‐6, IL‐10, and TNF‐α in the moderate‐to‐severe group were significantly higher, while the MoCA score was significantly lower. Multivariate linear regression analysis indicated that the NAFLD grade could significantly affect the MoCA score and the levels of IL‐1β and TNF‐α. Mediation analysis revealed that the level of TNF‐α significantly mediated the relationship between NAFLD and cognitive function, with a mediation effect size of 0.36.

**Conclusion:**

Elevated TNF‐α levels may be a key intermediate factor in the development of PSCI in patients with NAFLD. The prevention and treatment of NAFLD and the inhibition of the peripheral inflammatory response may lead to new targets for early screening and intervention in PSCI.

## Graphical abstract

This article reveals the mediating role of cytokine levels between non‐alcoholic fatty liver disease and post‐stroke cognitive impairment, providing new therapeutic targets for post‐stroke cognitive dysfunction.

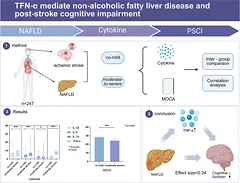

## 1 Introduction

Nonalcoholic fatty liver disease (NAFLD) is a globally prevalent chronic condition and has become an increasingly serious public health issue (Wang et al. 2022). NAFLD is closely related to inflammation. To date, many experiments have demonstrated that the levels of proinflammatory cytokines in the peripheral blood of patients with NAFLD are significantly elevated (Mirea et al. 2020; Wong et al. 2018). Because of the accumulation of lipids in NAFLD hepatocytes, the resulting lipotoxicity and oxidative stress induce the massive release of proinflammatory cytokines, which in turn promote the progression of NAFLD, forming a vicious cycle (El Amrousy and El‐Afify 2020).

In clinical practice, we have reported that NAFLD shares the same risk factors as those associated with cognitive dysfunction, such as diabetes, obesity, and hyperlipidemia (Bertolotti et al. 2014; Colognesi et al. 2020); therefore, we believe that NAFLD may have a negative effect on cognitive function. Recent studies have reported that patients with NAFLD perform significantly worse in terms of cognitive domains such as memory, mental speed, attention, psychomotor speed, and visual‐spatial perception (George et al. 2022). A cross‐sectional study in Japan also revealed that the scores of patients with NAFLD in a verbal fluency task (VFL) were significantly lower (Takahashi et al. 2017). Although the above studies have shown that NAFLD is associated with cognitive decline to some extent, there are still no relevant reports on whether NAFLD is involved in the pathogenesis of poststroke cognitive impairment (PSCI).

The levels of proinflammatory cytokines in the peripheral blood of patients with NAFLD are elevated (El Amrousy and El‐Afify 2020), and the levels of proinflammatory cytokines have proven to be significantly correlated with PSCI. Therefore, we speculate that proinflammatory cytokines may, to some extent, mediate the association between NAFLD and PSCI. In this study, we investigated the relationships among NAFLD, peripheral blood cytokine levels, and cognitive dysfunction after ischemic stroke. We further explored the mediating role of cytokine levels in this process, with the aim of identifying new targets for early screening and intervention in PSCI.

## 2 Materials and Methods

### 2.1 Research Subjects

A total of 247 patients with acute ischemic stroke who visited Xuzhou First People's Hospital from October 2022 to January 2025 were selected as the experimental group. They were divided into a no‐to‐mild group (146 cases) and a moderate‐to‐severe group (101 cases) according to the severity of NAFLD.

#### 2.1.1 Inclusion and Exclusion Criteria

The inclusion criteria for patients were as follows: (1) a clear diagnosis of acute ischemic cerebral infarction based on the diagnostic criteria for acute ischemic stroke in the guidelines (Chinese Neurology Society, Neurology Group of Cerebrovascular Disease of Chinese Medical Association 2018); (2) a first‐onset stroke; (3) the capability to undergo a cognitive function scale examination; and (4) the provision of informed consent for this study.

Patients were excluded if they met the following criteria: (1) undergoing thrombolytic or thrombectomy treatment; (2) liver diseases caused by long‐term alcohol abuse; (3) viral hepatitis, immune‐related liver diseases, and so on; (4) taking drugs that may affect liver metabolism within the last month, such as thiazides or glucocorticoids; (5) suffering from other autoimmune, tumor, or inflammatory diseases; (6) history of dementia, mild cognitive impairment, or other chronic conditions significantly affecting cognitive function; (7) having other organic diseases, such as neurological or mental disorders in the brain, apart from cerebral infarction; (8) having a history of substance abuse involving psychoactive substances; (9) unable to cooperate with the examination of cognitive function scales; and (10) patients with congenital or other cause‐induced intellectual disability.

### 2.2 Research Methodology

A detailed medical history was obtained for all patients, including name, sex, age, educational level, NIHSS score, infarction site, number of infarction lesions, classification of stroke causes, infarction area (with a boundary value set at the maximum diameter of 3.0 cm for acute infarction lesions), atrial fibrillation, hypertension, diabetes, smoking, alcohol consumption, coronary heart disease, hyperhomocysteinemia, cholesterol, triglycerides, low‐density lipoprotein, ALT, AST, and abdominal ultrasound examination findings. The levels of peripheral blood cytokines were measured in patients 3 months after stroke, and their cognitive function was evaluated. The clinical data, cognitive function scale scores, and cytokine levels of the no‐to‐mild group and the moderate‐to‐severe group were compared, and the correlations between the severity of NAFLD and cytokines, as well as the scale scores, were analyzed. The role of cytokines in the relationship between NAFLD and cognitive function was explored.

### 2.3 Assessment of Cognitive Function Scale

The Montreal cognitive assessment scale (MoCA) was used to evaluate the cognitive function of patients.

### 2.4 Cytokine Detection Methods

Venous blood (5 mL) was collected in the morning while the research subjects were fasting. The levels of IL‐1β, IL‐2, IL‐6, IL‐8, IL‐10, IL‐12, and TNF‐α were detected using the multiplex microsphere flow cytometry immunofluorescence luminescence method.

### 2.5 Diagnostic Criteria for Nonalcoholic Fatty Liver Disease

According to the “Diagnosis and Treatment Guidelines for Nonalcoholic Fatty Liver Disease (Revised Edition 2010)”, (1) the following clinical diagnostic criteria were met: (a) there was no history of alcohol consumption, or the amount of alcohol consumed was less than 70 g per week for women and less than 140 g per week for men; and (b) other causes were excluded, and the liver imaging diagnosis was consistent with diffuse fatty liver. (2) Ultrasound imaging diagnosis: (a) the near‐field echo of the liver was diffusely enhanced, and the echo was stronger than that of the kidneys; (b) the internal duct structures of the liver were unclear; and (c) the echo intensity in the far field of the liver area was decreased. If any two of these three criteria were met, a diagnosis of diffuse fatty liver was made.

The severity of NAFLD was classified by the examiners as none, mild, moderate, or severe. In the present study, we classified NAFLD into no‐to‐mild and moderate‐to‐severe NAFLD on the basis of previous studies (Seo et al. 2016).

### 2.6 Statistical Methods

Statistical analysis of the data was conducted using SPSS 26.0. The measurement data were subjected to normality tests. Data that met the normal distribution criteria are expressed as the mean ± standard deviation ($\bar{x}$±* s*). Intergroup comparisons were conducted using two independent samples t tests. Data that did not follow a normal distribution are expressed as *M* (*Q1, Q3*), and comparisons between the two groups were conducted using the Mann‒Whitney U test. The categorical count data are expressed as the number of cases (percentage), and comparisons between groups were conducted using the *χ*2 test. For the factors with statistical significance in the univariate analysis, multiple linear regression analysis was performed. The Process plugin was used to test the effects of peripheral blood cytokine levels on the relationship between NAFLD and cognitive function. A *p* value < 0.05 indicated a statistically significant difference.

## 3 Results

### 3.1 Basic Information and Comparison of MoCA Scores, as Well as Cytokine Levels

The Mann‒Whitney U test indicated that the levels of cholesterol, triglycerides, low‐density lipoprotein (LDL), ALT, AST, IL‐1β, IL‐6, IL‐10, and TNF‐α in the moderate‐to‐severe group were significantly higher than those in the no‐to‐mild group, while the MoCA score was significantly lower than that in the no‐to‐mild group. The results of the *χ* ^2^ test suggested that the prevalence of diabetes and hyperhomocysteinemia in the moderate‐to‐severe group was significantly higher than that in the no‐to‐mild group (*p* < 0.05). There were no statistically significant differences in age, sex, education level, NIHSS score, infarction site, number of infarction lesions, classification of stroke causes, infarction area, atrial fibrillation, hypertension status, smoking status, drinking status, coronary heart disease status, or levels of IL‐2, IL‐8, or IL‐12 between the two groups (*p > * 0.05), as shown in Table 1.

**TABLE 1:** Comparison of clinical data between the two groups.

	Moderate‐to‐severe	No‐to‐mild	X^2^/t/Z	*p*
Gender, n			0.025	0.874
Male	55(54.5%)	81(55.5%)		
Female	46(45.5%)	65(44.5%)		
Age	62.19 ± 12.05	62.60 ± 12.33	0.25	0.583
Educational level			1.41	0.495
Primary school	18(17.8%)	34(23.3%)		
Middle school	44(43.6%)	64(43.8%)		
High school and above	39(38.6%)	48(32.9%)		
NIHSS score	2.24 ± 1.01	2.54 ± 1.16	2.01	0.155
Infarction site			0.01	0.940
Superficial infarction	59(58.4%)	84(57.5%)		
Subtopic infarction	42(41.6%)	62(42.5%)		
The number of infarction lesions			2.293	0.130
Single lesion	83(82.2%)	108(74.0%)		
Multiple lesions	18(17.8%)	38(26.0%)		
TOAST			0.965	0.810
Atherosclerotic type of major arteries	51(50.5%)	72(49.3%)		
Cardiac type	8(7.9%)	17(11.6%)		
Small artery occlusion	28(27.7%)	39(26.7%)		
Other/Unexplained Type	14(13.9%)	18(12.3%)		
Infarction area			0.081	0.777
Small area (< 3.0)	62(61.4%)	87(59.6%)		
Medium to large area (≥ 3.0)	39(38.6%)	59(40.4%)		
Atrial fibrillation, n	8(101)	17(146)	0.91	0.340
Hypertension,n	79(101)	119(146)	0.41	0.524
Diabetes, n	59(101)	65(146)	4.61	0.032^*^
Smoking			5.49	0.064
No	49	51		
Previously	36	58		
Continuation	16	37		
Drinking alcohol			0.710	0.701
No	74	100		
Previously	24	40		
Continuation	3	6		
Coronary heart disease, n	17(101)	21(146)	0.28	0.600
Hyperhomocysteinemia, n	47(101)	34(146)	14.64	< 0.001^**^
Cholesterol	4.91(3.89,5.78)	4.04(3.06,5.25)	−3.57	< 0.001^**^
Triglyceride	1.89(1.25,2.84)	1.64(1.05,2.52)	−2.01	0.045^*^
LDL	3.37(2.82,3.95)	2.83(2.37,3.60)	−3.36	< 0.001^**^
ALT	35.40 ± 22.93	30.16 ± 16.37	−2.10	0.037^*^
AST	32.39 ± 14.83	27.56 ± 11.83	−2.84	0.005^**^
IL‐1β	7.83(3.21,12.38)	4.23(2.09,7.48)	−4.78	< 0.001^**^
IL‐2	2.53(1.54,3.78)	2.02(1.19,3.64)	−1.88	0.060
IL‐6	5.41(3.49,7.99)	3.24(1.89,5.38)	−4.92	< 0.001^**^
IL‐8	2.44(1.43,5.80)	2.12(1.43,3.86)	−1.59	0.113
IL‐10	1.53(1.01,2.52)	2.04(1.23,3.02)	−2.38	0.017^*^
IL‐12	1.83(1.23,3.02)	2.04(1.23,3.55)	−0.86	0.931
TNF‐α	7.68(6.04,9.53)	2.47(1.41, 3.76)	−11.35	< 0.001^**^
MoCA score	24.24 ± 1.99	28.19 ± 1.50	17.81	< 0.001^**^

^*^ *p*  < 0.05, ^**^ *p *< 0.01.

### 3.2 Multivariate Linear Regression Analysis of NAFLD and MoCA Scores

Using the MoCA score as the dependent variable and the NAFLD grade as the independent variable, a multiple linear regression analysis model was constructed. After adjusting for confounding factors such as age, sex, education level, diabetes status, hyperhomocysteinemia, and levels of cholesterol, triglycerides, low‐density lipoprotein, ALT, AST, IL‐1β, IL‐6, IL‐10, and TNF‐α, the NAFLD grade significantly affected the MoCA score (*p* < 0.05), as shown in Table 2.

**TABLE 2:** Multivariate linear regression analysis of non‐alcoholic fatty liver disease and MoCA scale scores.

	MOCA	
	β	*p*
Model 1	−0.771	< 0.001
Model 2	−0.741	< 0.001
Model 3	−0.486	< 0.001

*Note*: Model 1 adjusted for age, sex, and educational level. Model 2 adjusted for age, sex, education level, diabetes, hyperhomocysteinemia, cholesterol, triglycerides, LDL, ALT, and AST. Model 3 adjusted for age, sex, education level, diabetes, hyperhomocysteinemia, cholesterol, triglycerides, LDL, ALT, AST, IL‐1β, IL‐6, IL‐10, and TNF‐α.

### 3.3 Multivariate Linear Regression Analysis of NAFLD and Peripheral Blood Cytokine Levels

Using cytokine levels as the dependent variable and NAFLD grade as the independent variable, a multiple linear regression analysis model was constructed. After adjusting for confounding factors such as age, sex, education level, diabetes status, hyperhomocysteinemia status, and levels of cholesterol, triglycerides, low‐density lipoprotein, ALT, and AST, the NAFLD grade significantly affected the levels of IL‐1β and TNF‐α (*p* < 0.05), as shown in Table 3.

**TABLE 3:** Multivariate linear regression analysis of non‐alcoholic fatty liver disease and peripheral blood cytokine levels.

	β		
	Model 1	Model 2	Model 3
IL‐1β	0.294 ^* *^	0.309 ^* *^	0.222 ^* *^
IL‐6	0.143 ^*^	0.119	0.116
IL‐10	−0.063	−0.061	−0.050
TNF‐α	0.645 ^* *^	0.644 ^* *^	0.593 ^* *^

*Note*: Model 1 adjusted for age, sex, and educational level. Model 2 adjusted for age, sex, education level, diabetes, and hyperhomocysteinemia. Model 3 adjusted for age, sex, education level, diabetes, hyperhomocysteinemia, cholesterol, triglycerides, LDL, ALT, and AST.

^*^ *p * < 0.05, ^**^ *p *< 0.01.

### 3.4 Mediation Analysis

Taking the classification of NAFLD as the independent variable, the levels of the peripheral blood cytokines IL‐1β and TNF‐α as the mediating variables, and the MoCA score as the dependent variable, a mediation analysis was conducted. The results of the mediation analysis indicated that the level of TNF‐α significantly mediated the relationship between NAFLD and cognitive function, suggesting that NAFLD might cause changes in cognitive function by affecting the level of TNF‐α. IL‐1β did not significantly affect the interaction between the two (*p* > 0.05), as shown in Table 4 and Figure 1.

**TABLE 4:** Analysis of the mediating role of non‐alcoholic fatty liver disease, TNF‐α levels, and MoCA scale scores.

	Effect value	se	LLCT	ULCT	Effect size
overall effect	−4.05	0.21	−4.47	−3.63	
direct effect	−2.59	0.24	−3.07	−2.12	0.64
Mediating effect	−1.46	0.25	−2.09	−1.11	0.36

**FIGURE 1:**
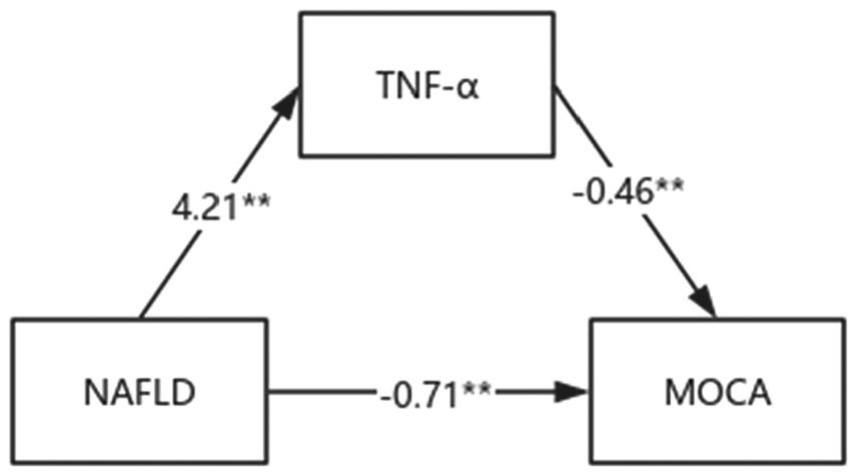
The mediating model diagram between nonalcoholic fatty liver disease, TNF‐α, and poststroke cognitive impairment.

## 4 Discussion

Numerous studies have shown that patients with NAFLD have a persistent low‐level inflammatory response in their peripheral blood (He et al. 2022; Duan et al. 2022). Some scholars have reported that in the adipose tissue of patients with NAFLD, the levels of proinflammatory immune cells are significantly elevated, and these cells continuously secrete inflammatory cytokines into the bloodstream (Binvignat et al. 2024). In this study, the levels of cytokines in the no‐to‐mild group and the moderate‐to‐severe group were compared, and the levels of IL‐1β, IL‐6, IL‐10, and TNF‐α in the moderate‐to‐severe group were significantly higher than those in the no‐to‐mild group. In addition, a multiple linear regression model revealed that the severity of NAFLD significantly affected the levels of proinflammatory cytokines in the peripheral blood, and as the severity increased, the levels of proinflammatory cytokines also increased. These findings further confirmed the close relationship between NAFLD and the inflammatory response. Fuchs et al. (2021) reported similar findings. Comparisons of the levels of subcutaneous adipose tissue and plasma inflammatory factors between patients with NAFLD and healthy controls revealed that the levels of these inflammatory markers, including total macrophages and proinflammatory (M1‐like) macrophages, activated and memory T‐cell subsets, and proinflammatory cytokines, were often significantly higher in the NAFLD group than in the healthy control group (Fuchs et al. 2021). Fontes‐Cal et al. (2021) reported that cytokines may be involved in the immune pathogenesis of NAFLD. According to the “double hit” hypothesis, insulin resistance, oxidative stress, and immune cell activation are the key pathogenic factors involved in the progression from simple steatosis to steatohepatitis. This theory suggests that lipid accumulation leads to liver steatosis, followed by multiple types of damage, including the secretion of adipokines, inflammatory responses, lipotoxicity, glucose and lipid metabolism disorders, and so on, ultimately triggering NASH and cirrhosis (Auguet et al. 2020). Proinflammatory cytokines, such as IL‐6, IL‐12, TNF, and IL‐10, are considered to play a central role in these processes (Stojsavljević et al. 2014). Some studies have shown that the plasma concentration of IL‐6 may be positively correlated with systemic insulin resistance, liver IL‐6 expression, liver inflammation, and the degree of liver fibrosis in patients with NAFLD (Fontes‐Cal et al. 2021). IL‐17 has also been shown to promote the progression of NASH and liver fibrosis (He et al. 2022). Certain cytokines, such as IL‐6, IL‐8, and adiponectin, may also be associated with the severity of NAFLD (Auguet et al. 2020; Damavandi and Zeinali 2021).

Fifty million people worldwide suffer from dementia (Fontes‐Cal et al. 2021). As the prevalence of cognitive impairment and dementia continues to increase, it is estimated that by 2050, the prevalence of cognitive impairment and dementia among older adults (aged 60 and above) will increase to approximately 2 billion, accounting for 22% of the global population (Takahashi et al. 2017). With the aging population and the lack of effective preventive intervention measures, the social and economic burden of these conditions has increased worldwide (Pérez Palmer et al. 2022). Early assessment and prevention of related risk factors are crucial for optimizing the management of cognitive dysfunction. In recent years, numerous studies have shown that NAFLD is closely associated with the onset of cognitive impairment (Hadjihambi et al. 2023; Wernberg et al. 2023). A cross‐sectional study involving 320 patients who underwent a liver biopsy revealed that compared with control patients, patients with NAFLD had poorer immediate and delayed memory. Severe hepatic steatosis is associated with increased memory impairment (Miao et al. 2023). A meta‐analysis revealed that compared with that in healthy controls, the risk of cognitive impairment in patients with NAFLD increased by a factor of 1.44 overall (Wang et al. 2022). In our study, compared with those in the no‐to‐mild group, the MoCA scores in the moderate‐to‐severe group were significantly lower. We then further conducted a multiple linear regression analysis using the MoCA score as the dependent variable and the severity of NAFLD as the independent variable. The results revealed that a higher severity of NAFLD was associated with a lower MoCA score. NAFLD had a significant negative effect on cognitive function, suggesting that NAFLD might also play an indispensable role in the pathogenesis of PSCI.

Proinflammatory cytokines may be involved in the pathogenesis of PSCI. In a mouse model of left unilateral common carotid artery occlusion (UCCAO) cerebral ischemia model, it was found that the levels of the proinflammatory cytokines TNF‐α and IL‐1β in the central nervous system significantly increased after acute ischemic stroke. This may lead to the expansion of brain damage and neuronal death and result in significant impairment of cognitive performance in mice (Silva et al. 2015). In clinical studies, Kulesh et al. (2018) reported significant differences in cytokine expression levels between patients with normal cognition and those with cognitive impairment after acute ischemic stroke. Serum IL‐6 levels and the levels of IL‐1β and IL‐10 in the cerebrospinal fluid were significantly higher in patients with executive dysfunction than in patients without cognitive impairment (Kulesh et al. 2018). Accumulating evidence suggests that cytokines may be related to neurogenesis, synaptic plasticity, long‐term potentiation (LTP), and the formation and consolidation of memory. This might be an important mechanism through which cytokines affect cognitive function (Silva et al. 2015). The levels of proinflammatory cytokines in the peripheral blood of patients with NAFLD are elevated, and these proinflammatory cytokines may also be involved in the pathogenesis of PSCI; thus, we reasonably speculate that cytokines may play a crucial role in the process through which NAFLD can damage cognitive function. Therefore, through mediation effect analysis, we found that TNF‐α plays a significant mediating role between NAFLD and MoCA scores. It is worth noting that the mediation analysis conducted in the present study is purely exploratory. Since cytokine levels and the MOCA scale were measured and assessed concurrently, there is currently a lack of longitudinal time‐series evidence to verify the causal relationships among the exposure variables, the mediating variables, and the outcome variables. Accordingly, the significant mediation effect identified in this study only confirms the statistical association between NAFLD, elevated TNF‐α, and cognitive decline, but does not establish a clear biological causal pathway. Inferring a robust biological causal pathway requires further validation via prospective longitudinal studies and mechanistic experiments. Notwithstanding this limitation, this exploratory mediation analysis still offers a novel perspective for elucidating the relationship between NAFLD and PSCI. Preventing and treating NAFLD and inhibiting peripheral inflammatory responses may lead to new targets for early screening and intervention in PSCI.

Recent animal studies have shown that TNF‐α antagonists can have beneficial effects on cognitive function (Ignatowski et al. 2014). Etanercept is a biological TNF‐α antagonist. It has been reported to reduce microglial cell activation in experimental models and can also reduce inflammation and vascular dysfunction, thereby improving cognitive deficits in rats (Gocmez et al. 2020). In some studies using animal models, researchers reported that mice treated with etanercept showed significant improvement in cognitive function (Tuttolomondo et al. 2014). However, at present, there is a lack of relevant clinical studies. In the future, more clinical experiments are needed to further verify the clinical therapeutic value of TNF‐α antagonists for PSCI.

## 5 Conclusion

This study confirmed that elevated TNF‐α levels may be a key intermediate factor in the development of PSCI in patients with NAFLD. Preventing and treating NAFLD and inhibiting the peripheral inflammatory response may lead to new targets for early screening and intervention in PSCI. The limitations of this study include the relatively small sample size and the single‐center design, which may not represent the general population of patients with ischemic stroke. In the future, multicenter studies should be conducted to expand the sample size and further verify the conclusions of this study. The measurement of cytokine levels and the assessment using the MoCA scale were conducted at the same time point; subsequent studies at different time points can be carried out to verify the causal relationship. In addition, the follow‐up time for assessing the cognitive function of patients with ischemic stroke in this study was relatively short. Future studies should extend the follow‐up time to explore whether these conclusions still hold.

## Author Contributions

**Xin Du**: conceptualization, methodology, data curation, investigation, writing – original draft. **Jingwei Li**: supervision, funding acquisition, resources, writing – review and editing. All authors have read and agreed to the published version of the manuscript.

## Funding

This work was supported by grants from the 2023 Key Medical Research Project of the Health Commission of Jiangsu Province: “Research on Risk Prediction Model and Treatment System for Symptomatic Intracranial Atherosclerotic Occlusion: A Multicenter, Randomized, Controlled Study” (H2023048).

## Ethics Statement

Ethical approval for this study was obtained from the Medical Ethics Committee of Xuzhou Municipal Hospital affiliated to Xuzhou Medical University {approval number:xyyll(2022)054}. All subjects knew the purpose of the study in advance and signed the informed consent form.

## Consent

Informed consent and permission to publish their information were obtained from the patients.

## Conflicts of Interest

The authors declare no conflicts of interest.

## Data Availability

The authors will supply the relevant data in response to reasonable requests.

## References

[brb371725-bib-0001] Auguet, T. , L. Bertran , J. Binetti , et al. 2020. “Relationship Between IL‐8 Circulating Levels and TLR2 Hepatic Expression in Women With Morbid Obesity and Nonalcoholic Steatohepatitis.” International Journal of Molecular Sciences 21, no. 11: 4189. 10.3390/ijms21114189. PMC7312372 32545403

[brb371725-bib-0002] Bertolotti, M. , A. Lonardo , C. Mussi , et al. 2014. “Nonalcoholic Fatty Liver Disease and Aging: Epidemiology to Management.” World Journal of Gastroenterology 20, no. 39: 14185. 10.3748/wjg.v20.i39.14185. PMC4202348 25339806

[brb371725-bib-0003] Binvignat, M. , J. Sellam , F. Berenbaum , and D. T. Felson . 2024. “The Role of Obesity and Adipose Tissue Dysfunction in Osteoarthritis Pain.” Nature Reviews Rheumatology 20, no. 9: 565–584. 10.1038/s41584-024-01143-3. 39112603

[brb371725-bib-0004] Chinese Neurology Society, Neurology Group of Cerebrovascular Disease of Chinese Medical Association . 2018. “Guidelines for the Diagnosis and Treatment of Acute Ischemic Stroke in China.” Chinese Journal of Neurology 51, no. 9: 666–682.

[brb371725-bib-0005] Colognesi, M. , D. Gabbia , and S. De Martin . 2020. “Depression and Cognitive Impairment—Extrahepatic Manifestations of NAFLD and NASH.” Biomedicines 8, no. 7: 229. 10.3390/biomedicines8070229. PMC7400092 32708059

[brb371725-bib-0006] Damavandi, N. , and S. Zeinali . 2021. “Association of Xenobiotic‐Metabolizing Enzymes (GSTM1 and GSTT 1), and Pro‐Inflammatory Cytokines (TNF‐α and IL‐6) Genetic Polymorphisms With Non‐Alcoholic Fatty Liver Disease.” Molecular Biology Reports 48, no. 2: 1225–1231. 10.1007/s11033-021-06142-1. 33492571

[brb371725-bib-0007] Duan, Y. , X. Pan , J. Luo , et al. 2022. “Association of Inflammatory Cytokines With Non‐Alcoholic Fatty Liver Disease.” Frontiers in Immunology 13: 880298. 10.3389/fimmu.2022.880298. PMC9122097 35603224

[brb371725-bib-0008] El Amrousy, D. , and D. El‐Afify . 2020. “Osteocalcin and Osteoprotegerin Levels and Their Relationship With Adipokines and Proinflammatory Cytokines in Children With Nonalcoholic Fatty Liver Disease.” Cytokine 135: 155215. 10.1016/j.cyto.2020.155215 32731119

[brb371725-bib-0009] Fontes‐Cal, T. C. M. , R. T. Mattos , N. I. Medeiros , et al. 2021. “Crosstalk Between Plasma Cytokines, Inflammation, and Liver Damage as a New Strategy to Monitoring NAFLD Progression.” Frontiers in Immunology 12: 708959. 10.3389/fimmu.2021.708959. PMC8383065 34447378

[brb371725-bib-0010] Fuchs, A. , D. Samovski , G. I. Smith , et al. 2021. “Associations Among Adipose Tissue Immunology, Inflammation, Exosomes and Insulin Sensitivity in People With Obesity and Nonalcoholic Fatty Liver Disease.” Gastroenterology 161, no. 3: 968–981.e12. 10.1053/j.gastro.2021.05.008. PMC8900214 34004161

[brb371725-bib-0011] George, E. S. , S. Sood , R. M. Daly , and S.‐Y. Tan . 2022. “Is There an Association Between Non‐Alcoholic Fatty Liver Disease and Cognitive Function? A Systematic Review.” BMC Geriatrics 22, no. 1: 47. 10.1186/s12877-021-02721-w. PMC8753832 35016619

[brb371725-bib-0012] Gocmez, S. S. , Y. Yazir , G. Gacar , et al. 2020. “Etanercept Improves Aging‐Induced Cognitive Deficits by Reducing Inflammation and Vascular Dysfunction in Rats.” Physiology & Behavior 224: 113019. 10.1016/j.physbeh.2020.113019. 32569601

[brb371725-bib-0013] Hadjihambi, A. , C. Konstantinou , J. Klohs , et al. 2023. “Partial MCT1 Invalidation Protects Against Diet‐Induced Non‐Alcoholic Fatty Liver Disease and the Associated Brain Dysfunction.” Journal of Hepatology 78, no. 1: 180–190. 10.1016/j.jhep.2022.08.008. 35995127

[brb371725-bib-0014] He, S. , S. Cui , W. Song , et al. 2022. “Interleukin‐17 Weakens the NAFLD/NASH Process by Facilitating Intestinal Barrier Restoration Depending on the Gut Microbiota.” mBio 13, no. 2: e0368821. 10.1128/mbio.03688-21. PMC9040850 35266816

[brb371725-bib-0015] Ignatowski, T. A. , R. N. Spengler , K. M. Dhandapani , H. Folkersma , R. F. Butterworth , and E. Tobinick . 2014. “Perispinal Etanercept for Post‐Stroke Neurological and Cognitive Dysfunction: Scientific Rationale and Current Evidence.” CNS Drugs 28, no. 8: 679–697. 10.1007/s40263-014-0174-2. PMC4110406 24861337

[brb371725-bib-0016] Kulesh, A. , V. Drobakha , E. Kuklina , I. Nekrasova , and V. Shestakov . 2018. “Cytokine Response, Tract‐Specific Fractional Anisotropy, and Brain Morphometry in Post‐Stroke Cognitive Impairment.” Journal of Stroke and Cerebrovascular Diseases 27, no. 7: 1752–1759. 10.1016/j.jstrokecerebrovasdis.2018.02.004. 29610037

[brb371725-bib-0017] Miao, Y. , B. Zhang , X. Sun , et al. 2023. “The Presence and Severity of NAFLD are Associated With Cognitive Impairment and Hippocampal Damage.” Journal of Clinical Endocrinology & Metabolism 108, no. 12: 3239–3249. 10.1210/clinem/dgad352. 37310344

[brb371725-bib-0018] Mirea, A.‐M. , R. Stienstra , T.‐D. Kanneganti , et al. 2020. “Mice Deficient in the IL‐1β Activation Genes Prtn3, Elane, and Casp1 are Protected Against the Development of Obesity‐Induced NAFLD.” Inflammation 43, no. 3: 1054–1064. 10.1007/s10753-020-01190-4. PMC7280336 32002713

[brb371725-bib-0019] Pérez Palmer, N. , B. Trejo Ortega , and P. Joshi . 2022. “Cognitive Impairment in Older Adults: Epidemiology, Diagnosis, and Treatment.” Psychiatric Clinics of North America 45, no. 4: 639–661. 10.1016/j.psc.2022.07.010 36396270

[brb371725-bib-0020] Seo, S. W. , R. F. Gottesman , J. M. Clark , et al. 2016. “Nonalcoholic Fatty Liver Disease is Associated With Cognitive Function in Adults.” Neurology 86, no. 12: 1136–1142. 10.1212/WNL.0000000000002498. PMC4820136 26911638

[brb371725-bib-0021] Silva, B. , L. Sousa , A. Miranda , et al. 2015. “Memory Deficit Associated With Increased Brain Proinflammatory Cytokine Levels and Neurodegeneration in Acute Ischemic Stroke.” Arquivos de Neuro‐Psiquiatria 73, no. 8: 655–659. 10.1590/0004-282X20150083. 26222355

[brb371725-bib-0022] Stojsavljević, S. , M. Gomerčić Palčić , L. Virović Jukić , L. Smirčić Duvnjak , and M. Duvnjak . 2014. “Adipokines and Proinflammatory Cytokines, the Key Mediators in the Pathogenesis of Nonalcoholic Fatty Liver Disease.” World Journal of Gastroenterology 20, no. 48: 18070. 10.3748/wjg.v20.i48.18070. PMC4277948 25561778

[brb371725-bib-0023] Takahashi, A. , S. Kono , A. Wada , et al. 2017. “Reduced Brain Activity in Female Patients With Non‐Alcoholic Fatty Liver Disease as Measured by Near‐Infrared Spectroscopy.” PLoS ONE 12, no. 4: e0174169. 10.1371/journal.pone.0174169. PMC5380307 28376101

[brb371725-bib-0024] Tuttolomondo, A. , R. Pecoraro , and A. Pinto . 2014. “Studies of Selective TNF Inhibitors in the Treatment of Brain Injury From Stroke and Trauma: A Review of the Evidence to Date.” Drug Design, Development and Therapy 8: 2221. 10.2147/DDDT.S67655. PMC4232043 25422582

[brb371725-bib-0025] Wang, L. , B. Sang , and Z. Zheng . 2022. “Risk of Dementia or Cognitive Impairment in Non‐Alcoholic Fatty Liver Disease: A Systematic Review and Meta‐Analysis.” Frontiers in Aging Neuroscience 14: 985109. 10.3389/fnagi.2022.985109. PMC9530447 36204558

[brb371725-bib-0026] Wang, Y. , C. Chen , J. Chen , et al. 2022. “Overexpression of NAG‐1/GDF15 Prevents Hepatic Steatosis Through Inhibiting Oxidative Stress‐Mediated dsDNA Release and AIM2 Inflammasome Activation.” Redox Biology 52: 102322. 10.1016/j.redox.2022.102322. PMC9079118 35504134

[brb371725-bib-0027] Wernberg, C. W. , L. L. Grønkjær , B. Gade Jacobsen , et al. 2023. “The Prevalence and Risk Factors for Cognitive Impairment in Obesity and NAFLD.” Hepatology Communications 7, no. 7: e00203. 10.1097/HC9.0000000000000203. PMC10309508 37378627

[brb371725-bib-0028] Wong, V. W.‐S. , L. A. Adams , V. de Lédinghen , G. L.‐H. Wong , and S. Sookoian . 2018. “Noninvasive Biomarkers in NAFLD and NASH — Current Progress and Future Promise.” Nature Reviews Gastroenterology & Hepatology 15, no. 8: 461–478. 10.1038/s41575-018-0014-9. 29844588

